# Supplementary material for: In Silico Study of the Acquired Resistance Caused by the Secondary Mutations of KRAS G12C Protein Using Long Time Molecular Dynamics Simulation and Markov State Model Analysis
Source: Int J Mol Sci. 2022 Nov 10;23(22):13845. doi: 10.3390/ijms232213845 (PMC9694466; doi:10.3390/ijms232213845)
Supplement: Supplementary file 1 [file ijms-23-13845-s001.zip › ijms-1977134-supplementary.pdf]

Supporting information

In silico Study of the Acquired Resistance Caused by the Secondary Mutations of KRAS G12C Protein Using Long Time Molecular Dynamics Simulation and Markov State Model Analysis

Gao Tu[1,†], Qing Liu[1,†], Yue Qiu[1], Elaine Lai-Han Leung[2,*], Xiaojun Yao[2,*]

[1]*Dr. Neher's Biophysics Laboratory for Innovative Drug Discovery, State Key Laboratory of Quality Research in Chinese Medicine, Macau Institute for Applied Research in Medicine and Health, Macau University of Science and Technology, Taipa, Macau, China*

[2]*Cancer Center, Faculty of Health Science, University of Macau, Macau (SAR), China. MOE Frontiers Science Center for Precision Oncology, University of Macau, Macau (SAR), China*

[*] *Correspondence: lhleung@um.edu.mo; xjyao@must.edu.mo;*

[†] *These authors contributed equally to this work.*

Table S1. Energy components for the binding of AMG510 to the mutated KRAS.

System	Residues	AMG510			
		van der Waals	Electrostatic	Polar Solvation	Non-Polar Solvation
G12C	K16	-0.59	-3.70	2.22	-0.06
	R68	-1.38	2.20	-1.44	-0.14
	Y96	-4.08	-1.19	2.42	-0.47
G12C-K16T	K16T	-0.13	0.21	-0.14	0.00
G12C-R68S	R68S	-0.39	-0.05	0.30	-0.04
G12C-Y96C	Y96C	-1.34	-0.42	0.76	-0.13
G12C-Y96D	Y96D	-1.29	1.56	0.84	-0.16

Table S2. Energy components for the binding of MRTX849 to the mutated KRAS.

System	Residues	MRTX849			
		van der Waals	Electrostatic	Polar Solvation	Polar Solvation
G12C	K16	-1.08	12.82	-11.66	-0.08
	R68	-1.70	15.92	-14.99	-0.14
	Y96	-3.49	0.08	1.46	-0.39
G12C-K16T	K16T	-0.16	0.39	-0.31	0.00
G12C-R68S	R68S	-0.51	0.11	0.20	-0.04
G12C-Y96C	Y96C	-1.24	-0.34	0.78	-0.08
G12C-Y96D	Y96D	-1.37	-16.47	18.43	-0.12

Table S3. Calculated binding free energies for GDP binding in apo and inhibitor bound to mutated KRAS G12C, G12C-R68, G12C-K16T, G12C-Y96C and G12C-Y96D, respectively.

System		GDP		
		$\Delta G_{MM/GBSA}$	$-T\Delta S$	ΔG_{cal}
Apo	G12C	−115.07±11.03	40.35±4.41	−74.72
	G12C-R68S	−95.56±11.78	35.33±4.84	−60.23
	G12C-K16T	−71.26±13.39	35.53±4.60	−35.53
	G12C-Y96C	−115.54±20.88	41.25±4.96	−74.29
	G12C-Y96D	−118.03±11.99	40.80±5.57	−77.23
AMG510	G12C	−119.37±10.04	46.02±4.91	−73.35
	G12C-R68S	−117.70±9.59	40.98±4.73	−76.72
	G12C-K16T	−78.28±9.93	37.64±6.37	−40.64
	G12C-Y96C	−119.67±10.37	40.91±7.07	−78.76
	G12C-Y96D	−119.79±10.85	40.14±4.80	−79.65
MRTX849	G12C	−101.70±8.39	37.18±5.83	−64.52
	G12C-R68S	−99.97±8.27	36.59±5.57	−63.38
	G12C-K16T	−67.58±24.87	34.94±5.99	−32.64
	G12C-Y96C	−99.53±9.48	37.55±11.87	−61.98
	G12C-Y96D	−102.30±11.00	35.96±4.82	−66.34

Table S4. Fingerprints of molecular interaction including the interaction types between GDP and mutant APO KRAS protein.

Residues	Interaction type	Frequency (%)				
		G12C	G12C-R68S	G12C-K16T	G12C-Y96C	G12C-Y96D
G13	Hydrophobic	92.86	98.98	99.48	88.14	87.64
	HBAcceptor	73.08	91.80	82.98	70.34	72.64
V14	HBAcceptor	30.42	0.00	0.00	36.08	36.84
G15	Hydrophobic	99.98	99.98	99.92	99.82	99.94
	HBAcceptor	97.76	98.36	95.50	92.48	98.46
K16	HBAcceptor	99.96	100.00	99.96	94.76	99.98
	Anionic	100.0	100.00	0.00	95.04	100.00
S17	HBAcceptor	88.68	99.32	98.98	85.06	91.76
A18	Hydrophobic	99.50	99.78	99.84	99.42	99.46
	HBAcceptor	94.28	89.36	84.32	92.86	95.92
F28	Hydrophobic	94.54	97.18	91.92	97.78	95.06
	PiStacking	49.92	58.86	54.22	56.62	54.26
D30	Hydrophobic	33.28	48.90	40.08	34.92	38.20
	HBDonor	37.56	47.78	41.16	41.68	46.94
N116	Hydrophobic	52.68	56.10	60.32	50.70	53.94
K117	Hydrophobic	100.00	100.00	100.00	100.00	100.00
D119	Hydrophobic	99.96	99.94	99.94	99.98	99.94
	HBDonor	100.00	100.00	99.98	100.00	99.98
A146	Hydrophobic	91.70	93.54	92.86	91.48	91.02
	HBAcceptor	47.04	46.62	41.76	49.08	47.56
K147	Hydrophobic	97.90	97.96	97.34	96.88	96.84
	HBAcceptor	75.92	77.38	77.16	72.34	73.02

Table S5. Fingerprints of molecular interaction including the interaction types between GDP and mutant AMG150-bound KRAS complexes.

Residues	Interaction type	Frequency (%)				
		G12C	G12C-R68S	G12C-K16T	G12C-Y96C	G12C-Y96D
G13	Hydrophobic	80.72	90.52	98.08	83.20	91.22
	HBAcceptor	91.64	87.64	87.06	87.36	84.62
V14	HBAcceptor	39.30	46.22	31.54	42.48	34.46
G15	Hydrophobic	100.00	100.00	100.00	99.94	99.92
	HBAcceptor	98.64	98.48	95.44	98.76	98.30
K16	HBAcceptor	99.96	99.88	99.94	99.98	99.96
	Anionic	100.00	100.00	0.00	100.00	100.00
S17	HBAcceptor	85.62	94.06	98.80	84.42	87.72
A18	Hydrophobic	99.66	99.34	99.70	99.90	95.80
	HBAcceptor	97.36	95.24	84.56	97.92	89.18
F28	Hydrophobic	97.28	96.68	93.98	93.10	95.40
	PiStacking	52.94	54.48	52.62	49.64	51.46
V29	HBDonor	50.22	32.10	35.22	42.92	0.00
	HBDonor	0.00	37.64	0.00	0.00	0.00
D30	Hydrophobic	59.26	48.08	50.40	45.78	42.20
	HBDonor	63.86	52.34	53.70	50.32	45.46
N116	Hydrophobic	48.20	53.52	61.62	50.50	56.00
K117	Hydrophobic	100.00	100.00	100.00	100.00	100.00
D119	Hydrophobic	99.98	99.98	99.92	99.90	99.88
	HBDonor	100.00	100.00	100.00	99.94	100.00
A146	Hydrophobic	92.56	91.10	88.70	91.70	89.70
	HBAcceptor	53.34	44.78	37.24	49.08	41.04
K147	Hydrophobic	96.36	97.80	98.78	96.88	97.74
	HBAcceptor	67.88	75.50	82.60	70.38	77.22

Table S6. Fingerprints of molecular interaction including the interaction types between GDP and mutant MRTX849-bound KRAS complexes.

Residues	Interaction type	Frequency (%)				
		G12C	G12C-R68S	G12C-K16T	G12C-Y96C	G12C-Y96D
G13	Hydrophobic	99.28	98.72	98.64	98.38	99.42
	HBAcceptor	94.16	94.80	71.28	95.54	94.16
G15	Hydrophobic	99.96	100.00	99.98	100.00	100.00
	HBAcceptor	98.78	98.66	78.28	98.94	98.72
K16	HBAcceptor	99.98	99.98	82.16	100.00	100.00
	Anionic	100.00	100.00	86.62	100.00	100.00
S17	HBAcceptor	99.92	99.88	99.86	98.00	99.70
A18	Hydrophobic	100.00	99.98	0.00	99.94	99.88
	HBAcceptor	95.24	95.24	0.00	93.60	94.54
F28	Hydrophobic	97.88	98.96	97.96	98.56	98.30
	PiStacking	59.90	63.70	0.00	65.52	62.40
V29	Hydrophobic	60.14	54.42	33.98	44.50	33.38
	HBDonor	0.00	0.00	0.00	36.56	41.10
D30	Hydrophobic	44.08	54.48	33.10	59.12	60.34
	HBDonor	43.74	55.62	0.00	51.90	62.30
N116	Hydrophobic	58.18	57.30	100.00	57.88	58.54
K117	Hydrophobic	100.00	100.00	99.98	100.00	100.00
D119	Hydrophobic	99.92	99.98	0.00	99.98	99.92
	HBDonor	99.98	100.00	0.00	100.00	100.00
A146	Hydrophobic	92.84	92.88	0.00	92.48	92.96
	HBAcceptor	43.38	45.00	0.00	42.50	43.86
K147	Hydrophobic	96.56	96.42	99.24	96.82	96.54
	HBAcceptor	72.50	73.56	76.70	74.48	74.20

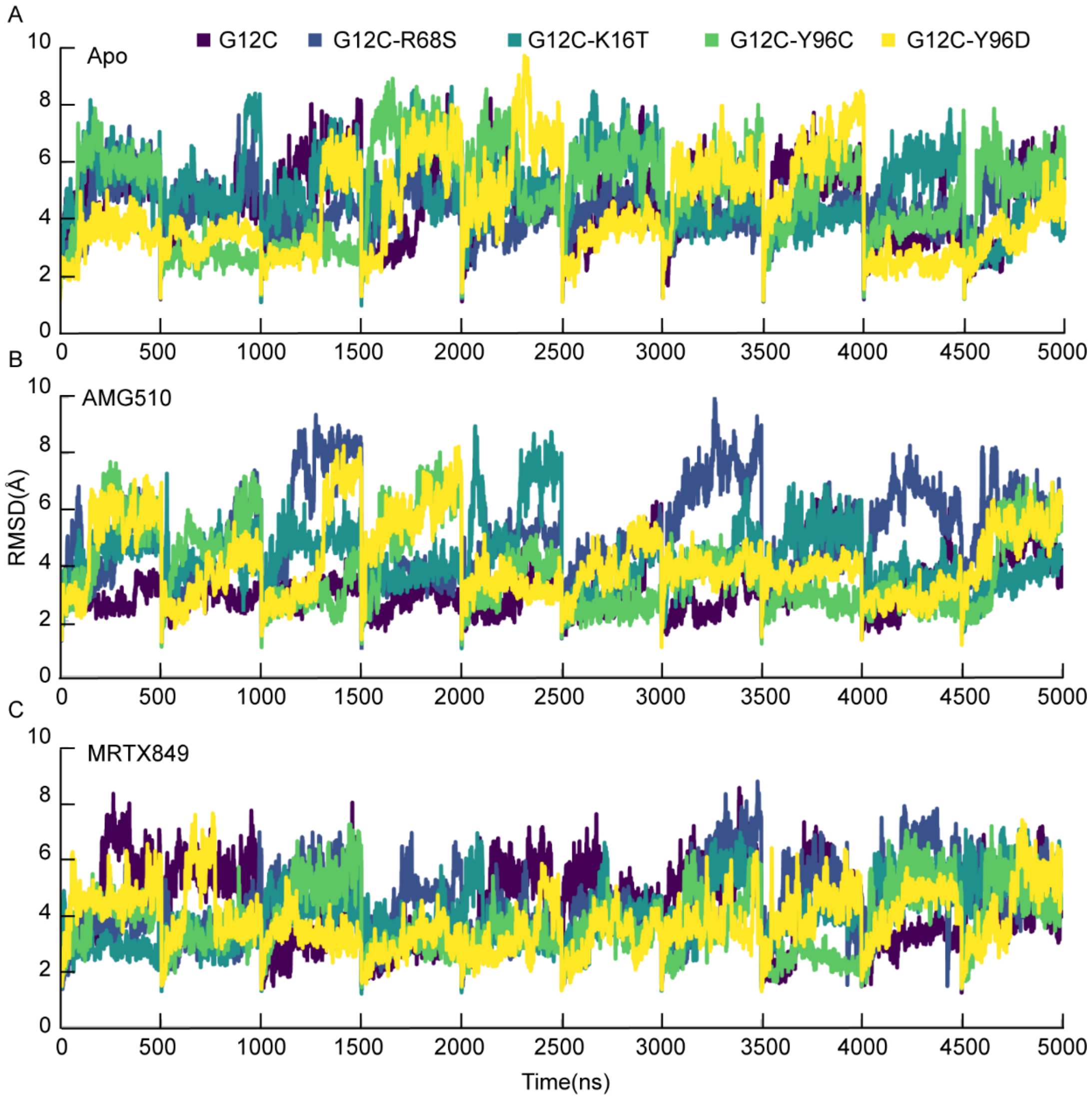

Figure S1. The monitored root-mean-square deviation (RMSD) of protein backbone atoms of the five apo, five AMG510-bound and five MRTX849-bound complexes during the MD simulation. (A-C) Ten independent MD simulations for each apo, AMG510-bound and MRTX849-bound complex, respectively.

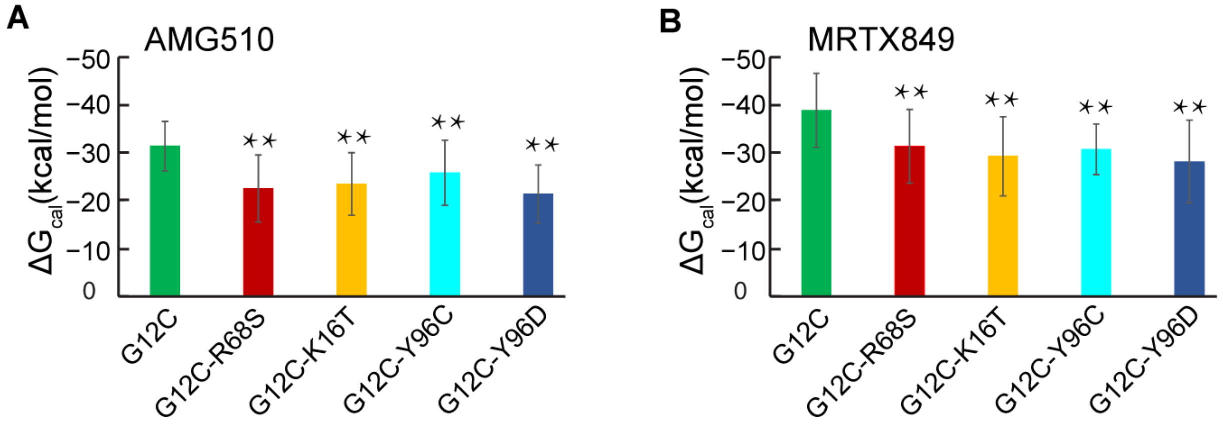

Figure S2. (A-B) The calculated binding free energy (ΔG_{cal}) for AMG510- and MRTX849-bound complexes. Mean±SD is shown. **p<0.01 versus KRAS G12C, t-test P-values indicate statistical significance (*P < 0.05 and **P < 0.01).

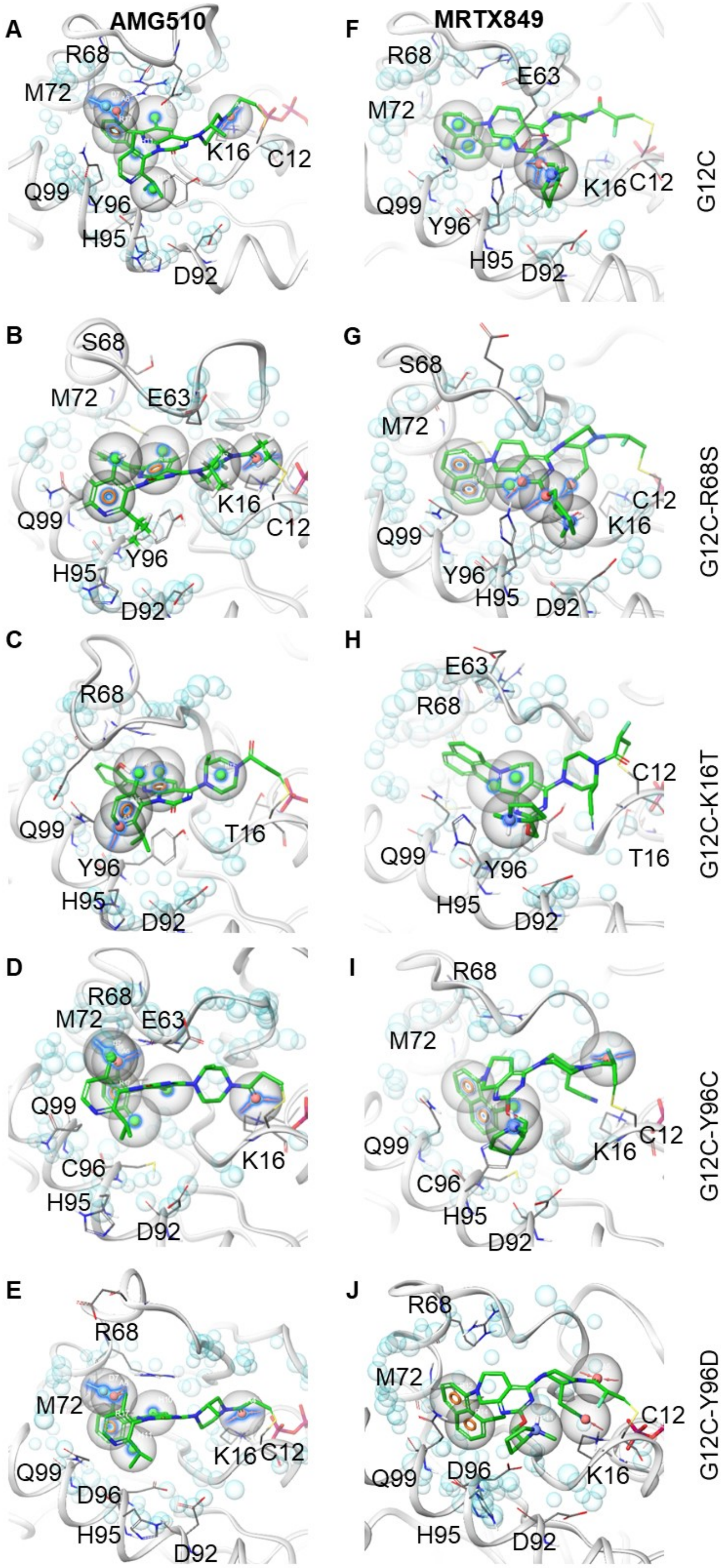

Figure S3. Pharmacophore models of AMG510(A-E) and MRTX849(F-J) binding to KRAS G12C,

G12C-R68S, G12C-K16T, G12C-Y96C and G12C-Y96D, respectively. The inhibitors in the binding site are shown as green sticks. The cyan spheres mean the excluded volume shell. Red arrows denote hydrogen bond acceptors, blue arrows denote donors, the orange ring indicates an aromatic ring, the green spheres denote hydrophobic centers the dark blue sphere indicates positively charged moiety.

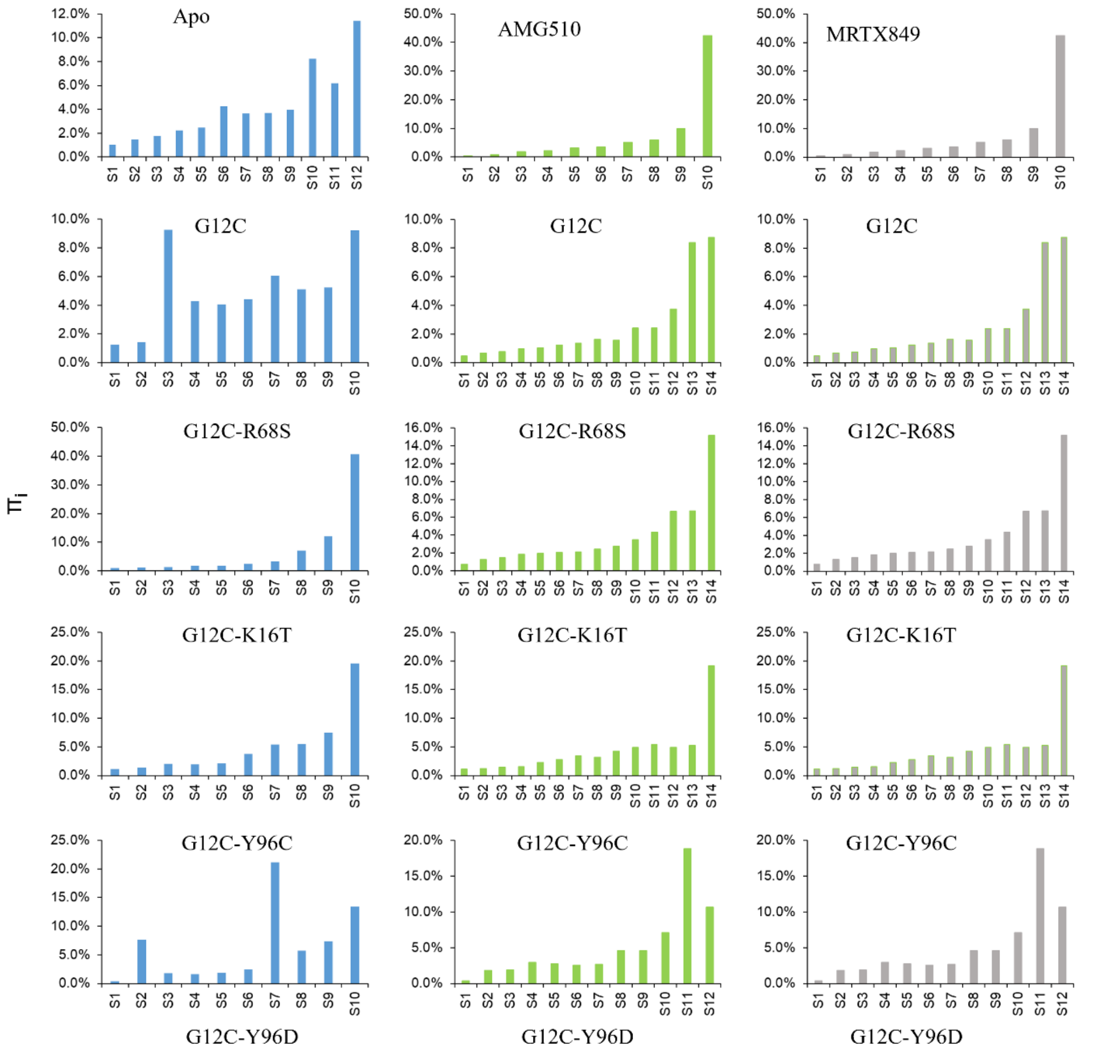

Figure S4. The equilibrium probability (π_i) of each metastable state in MSM. (First column) Probability (πi) of the apo form KRAS G12C and four second-site mutilation KRAS. Probability (πi) of the AMG510 (second column) and MRTX849(third column) bound to KRAS G12C and four second-site mutilation KRAS.

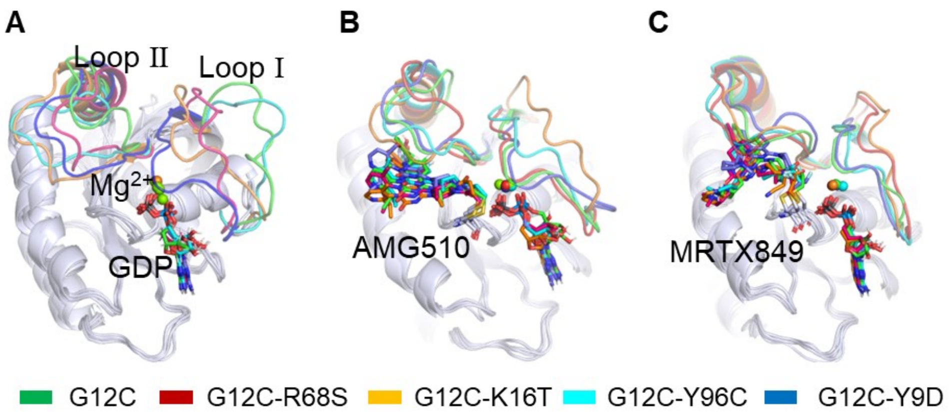

Figure S5. (A-C) Superposition of representative conformations for Apo, AMG510- and MRTX849-bound complexes. Each representative structure corresponding to the main low-energy basin.
